# An event-related potential study of spatial working memory in binge drinking college students

**DOI:** 10.1371/journal.pone.0203696

**Published:** 2018-09-10

**Authors:** Sunyoung Park, Myung-Sun Kim

**Affiliations:** Department of Psychology, Sungshin Women’s University, Seoul, South Korea; Universita degli Studi di Roma La Sapienza, ITALY

## Abstract

This study used event-related potentials (ERPs) and a modified spatial *2*-back task to investigate spatial working memory in binge drinking (BD) college students. Based on the Korean version of the Alcohol Use Disorder Identification Test (AUDIT-K) and Alcohol Use Questionnaire (AUQ) scores, participants were assigned into BD (*n* = 25) and non-BD (*n* = 25) groups. The modified spatial *2*-back task includes congruent, incongruent, and lure conditions and participants are required to respond as rapidly and accurately as possible to the congruent stimuli but not to the incongruent and lure stimuli. The BD and non-BD groups exhibited comparable performances on the spatial *2*-back task but the BD group showed significantly larger P3 amplitudes than the non-BD group. Additionally, the non-BD group showed larger P3 amplitudes in response to the congruent stimuli compared to the incongruent and lure stimuli whereas the P3 amplitudes in the BD group did not differ significantly among the three conditions. These results indicate that the BD individuals exerted greater effort to maintain performance levels comparable to non-BD individuals and that they were less efficient in differentiating or allocating attentional resources between relevant and irrelevant information.

## Introduction

Binge drinking (BD), a pattern of excessive alcohol consumption followed by a period of abstinence, is defined on the basis of the quantity, frequency and speed of alcohol consumption. For example, the National Institute on Alcohol Abuse and Alcoholism [1] defined BD as a pattern of drinking alcohol that brings blood alcohol concentration levels to 0.08 g/dL, which typically occurs after 4 drinks for women and 5 drinks for men in about 2 hours. BD, which has detrimental effects on health and social functioning [2–4], is particularly prevalent among college students [5,6]; it increases the likelihood of the development of an alcohol use disorder (AUD,[7]). Additionally, structural and functional brain abnormalities [8] and cognitive dysfunction [9,10] have been observed in individuals with BD.

Spatial working memory can be defined as the temporary maintenance and manipulation of spatial information [11,12] that affects higher cognitive functions such as inhibition, problem-solving, and goal-directed behavior [13,14]. Neuroimaging studies have revealed that the prefrontal and parietal cortices are involved in spatial working memory. For example, increased activity in the dorsolateral prefrontal cortex, posterior parietal cortex, and cerebellum is observed in normal controls during the performance of a spatial working memory task [15,16]. More specifically, the dorsolateral prefrontal cortex is involved in the online maintenance and manipulation of spatial information while the parietal cortex is involved in the storage of spatial information [17,18].

Because chronic alcohol consumption is associated with dysfunction of the prefrontal and parietal cortices, many studies have investigated spatial working memory deficits in patients with AUD [19–22]. Studies that investigated spatial working memory in patients with AUD using neuropsychological assessments revealed that AUD patients perform significantly less well than normal controls [23,24]. Additionally, AUD patients exhibit decreased activity in the bilateral dorsolateral prefrontal and parietal cortices while performing spatial working memory tasks [20,21,25].

The spatial *n*-back task is widely used to evaluate spatial working memory as it requires participants to determine whether a current stimulus is in the same or a different location as a stimulus presented *n* trials earlier [26,27]. Studies investigating spatial working memory using spatial *n*-back tasks have shown that AUD patients exhibit significantly lower accuracy than normal controls [21,28]. Several recent studies modified the original *n*-back task to assess interference control [29,30], which is defined as the ability to inhibit information that is irrelevant to the task, because it plays an important role in working memory [30,31]. The modified spatial *n*-back task is the same as the original task in that the participant is required to determine whether the location of the current stimulus is the same as that of the stimulus presented *n* trials earlier. However, the modified version also includes a lure condition along with the congruent (locations of current stimulus and that presented *n* trials earlier are the same) and incongruent (locations of current stimulus and that presented *n* trials earlier are different) conditions. In the lure condition, the same stimulus is immediately represented. If the same stimulus is immediately represented, participants can misidentify the lure stimulus as a congruent one, leading to an incorrect response [29,30].

Studies investigating spatial working memory in normal controls using the modified spatial *n*-back task have observed significantly longer response times and lower accuracy rates in response to lure stimuli than to incongruent stimuli [30]. These results indicate that the repeatedly presented identical stimuli produce interference while the successively presented stimuli are being updated by the working memory system, which would require greater cognitive effort to control the lure stimuli. Therefore, the modified *n*-back task is considered more appropriate for evaluating working memory than the original task because the modified task requires the participant to control the interference, which affects working memory performance [30,31].

Studies investigating spatial working memory in individuals with BD using neuropsychological tests have reported somewhat inconsistent findings. For example, some studies found significantly worse performance by BD individuals than by non-BD individuals [32,33] while others observed comparable performances [34,35].

Contrary to behavioral findings from working memory tasks, neuroimaging studies have consistently reported that BD individuals exhibit different patterns of cerebral activation compared to those of non-BD individuals while performing such tasks [36–38]. For example, Squeglia et al. [38] observed increased activation in the frontal and anterior cingulate cortices of male adolescents with BD relative to non-BD individuals during the performance of a spatial working memory task as well as significant positive correlations between the increased activation and performance. Studies investigating verbal working memory in BD individuals observed increased activation in the supplementary premotor area [36], right superior frontal cortex, and bilateral posterior parietal cortices [37] in BD individuals compared to non-BD individuals. Taken together, these findings support the compensation hypothesis; i.e., greater activation of cortical areas associated with working memory is required for BD individuals to maintain behavioral performance comparable to non-BD individuals in working memory tasks [36–38].

Although neuroimaging studies have identified the brain areas involved in spatial working memory, these findings provide limited information about the sequential stages of spatial working memory. Event-related potentials (ERPs) are widely used to assess cognitive function, including working memory, due to the high temporal resolution associated with this technique [39]. ERPs are electrical activities elicited by time-locked stimuli that contain certain information and consist of several peaks or components of positive and negative potentials. These peaks reflect the various stages of information processing [40].

Studies that employed the *n*-back task and examined ERPs have consistently identified two ERP components, N2 and P3, as important in working memory [27,41]. In normal controls, the amplitude of N2, which is a negative peak observed from 170–340 ms after stimulus-onset, is larger in response to incongruent stimuli than in response to congruent stimuli [42–44]. Additionally, a larger N2 amplitude is observed in response to lure stimuli than in response to incongruent stimuli [29,43]. The N2 reflects the inhibition of inappropriate responses [29,43] or detection processing, in which a current stimulus is judged to be congruent or incongruent with the one presented *n* trials earlier [42].

In normal controls, the amplitude of P3, which is a positive peak that occurs from 250~550 ms after stimulus-onset, is larger in response to congruent stimuli than it is in response to incongruent stimuli [45,46]. P3 reflects the classification of congruent/incongruent stimuli [27,47] and is associated with allocation of attention during stimulus encoding/maintenance [48] or memory updating [49–51].

Few studies have investigated working memory in AUD patients and BD individuals using ERPs. Patients with AUD exhibit significant reductions in P3 amplitudes compared to normal controls [52,53] and the ERP patterns of BD individuals differ from those of non-BD individuals [54–56]. For example, Crego et al. [55] found that BD individuals exhibited larger N2 amplitudes in response to congruent stimuli than did non-BD individuals. Furthermore, non-BD individuals had larger P3 amplitudes in response to congruent stimuli than in response to incongruent stimuli, whereas the P3 amplitudes of BD individuals did not differ between congruent and incongruent stimuli. Taken together, these results indicate that BD individuals have deficits in memory updating and exert greater cognitive effort to perform working memory tasks as successfully as non-BD individuals.

Thus, the present study examined spatial working memory in BD college students using ERPs and a modified spatial *n*-back task. Based on previous findings, it was hypothesized that the BD and non-BD groups would not differ significantly in terms of behavioral performance on the spatial working memory task but that the two groups would exhibit different ERP patterns. Based on previous findings, we hypothesized that BD individuals would exhibit larger N2 amplitudes in response to congruent stimuli than non-BD individuals. In addition, BD individuals would exhibit no differences in P3 amplitudes in response to the three types of stimuli whereas non-BD individuals would exhibit larger P3 amplitudes in response to congruent stimuli than in response to incongruent or lure stimuli. To our knowledge, no studies have investigated spatial working memory in BD individuals using ERPs and a modified *n*-back task.

## Materials and methods

### Ethics statement

The participants were instructed to abstain from alcohol consumption for 48 h prior to the experiment, and provided written informed consent after receiving a description of the study. The students were paid for their participation. This study was approved by the Sungshin Women’s University Institutional Review Board (SSWUIRB 2016–017).

### Participants

The detailed procedures of the participant selection process have been described previously in a study by our research group [57]. The Korean version of the Alcohol Use Disorder Identification Test (AUDIT-K, [58,59]), Alcohol Use Questionnaire (AUQ, [60]) and a questionnaire containing items about alcohol use (e.g., frequency of BD episodes in the last 2 weeks and age of onset of alcohol use) were administered to 310 college students. For this study, BD was defined based on the quantity, frequency, and speed of alcohol consumption; either four (female) or five (male) drinks in a short period of time more than once during the previous 2 weeks [61,62] and either two (female) or three (male) drinks per hour [1].

According to the World Health Organization (WHO), a score > 8 on the AUDIT is recommended as the cut-off point for problem drinking [58]. However, others have suggested that the sensitivity and specificity for problem drinking are highest when an AUDIT score >12 is used as a cut-off [59,63]. An AUDIT score > 26 indicates the possibility of alcohol dependence [63].

In this study, those who obtained total scores of 12~26 on the AUDIT-K, had drunk four (female) or five (male) glasses more than once during the previous 2 weeks, and drank more than two (female) or three (male) glasses per hour were included in the BD group. Those who obtained total scores < 8 on the AUDIT-K, had drunk less than four (female) or five (male) glasses during the previous 2 weeks, and drank less than two (female) or three (male) glasses per hour were included in the non-BD group.

Because parental alcohol use can influence the alcohol use of their offspring [64], the Korean version of the Children of Alcoholics Screening Test (CAST-K, [65,66]) was administered to identify whether participants’ parents had a history of AUD; those who obtained a score > 6 on the CAST-K were excluded from the present study. To control for levels of intelligence, anxiety, and depression, the Korean Wechsler Intelligence Scale (KWIS, [67]), Spielberger’s State-Trait Anxiety Inventory (STAI, [68]), and Self-Rating Depression Scale (SDS, [69]), respectively, were administered. Additionally, the Structured Clinical Interview for DSM-IV-Non Patient (SCID-NP, [70]) was administered to ensure that no participants had neurological, psychiatric disorders and drug/alcohol abuse.

Following the application of the initial inclusion and exclusion criteria, 46 students were placed in the BD group and 52 students were placed in the non-BD group. Subsequently, participants who were left handed, ambidextrous, had histories of neurological/ psychiatric disorders, or who refused to participate were also excluded from the study. Ultimately, there were 25 participants (8 males and 17 females) in both the BD (age range: 18–26 years) and non-BD (age range: 19–27 years) groups.

### The modified spatial 2-back task

A modified spatial *2*-back task was used to evaluate spatial working memory. This task included congruent, incongruent, and lure conditions; under the congruent condition, the locations of the current stimulus and the stimulus presented two trials earlier were the same; under the incongruent condition, the locations of the current stimulus and the stimulus presented two trials earlier were different; and under the lure condition, the stimulus presented one trial earlier was repeated. The stimulus, a red square, was presented in a 3 × 3 matrix and in total 360 trials (congruent, incongruent, and lure stimuli presented 108, 198, and 54 times, respectively) were administered in two blocks with the congruent, incongruent, and lure stimuli presented randomly. Participants were required to respond as accurately and quickly as possible in the congruent trials but not respond in the incongruent and lure trials.

**Fig 1:**
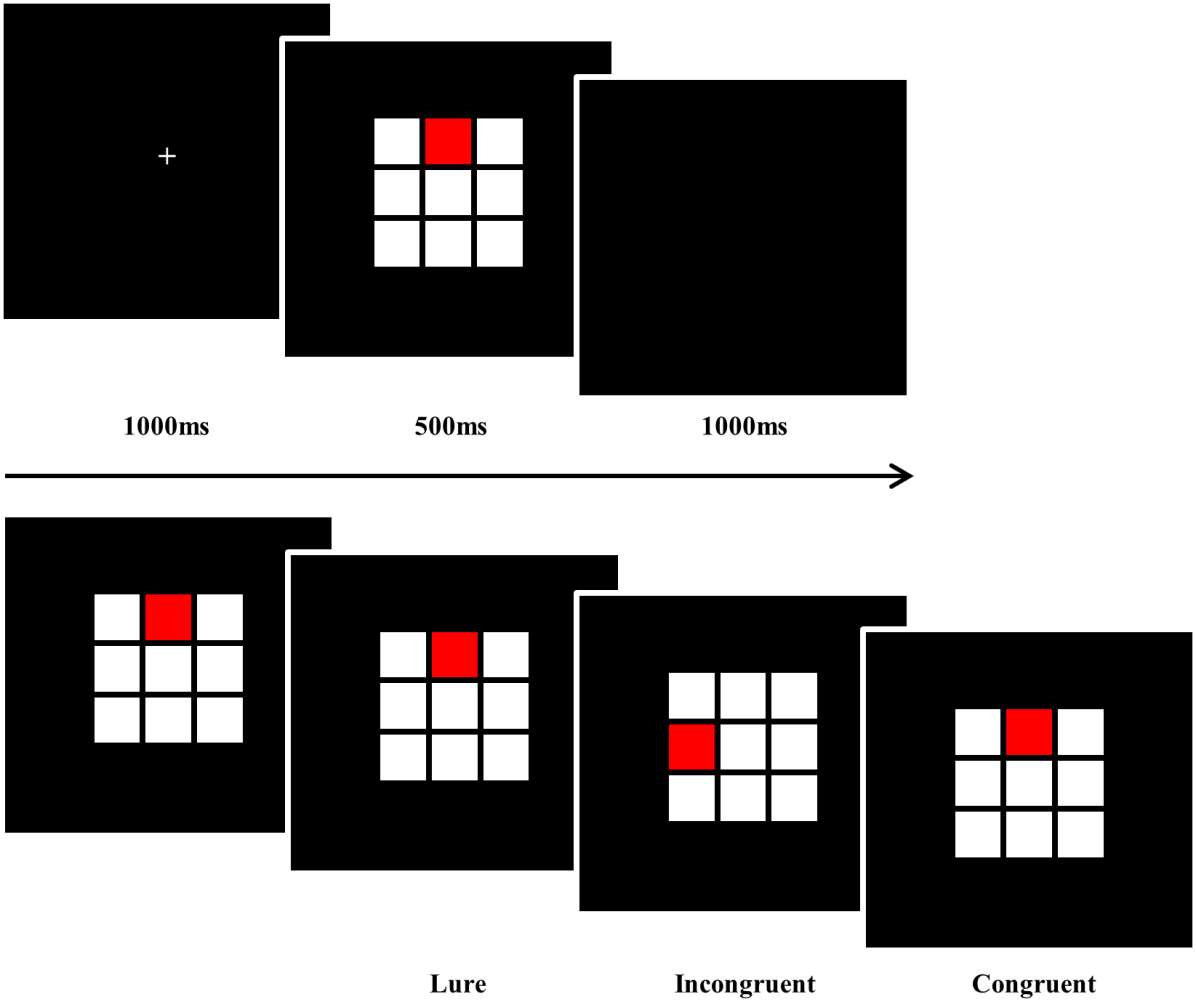
The spatial *2*-back task. The spatial *2*-back task consists of a congruent condition, under which the locations of the current stimulus and the stimulus presented 2 trials earlier are the same, incongruent condition, under which the locations of the current stimulus and the stimulus presented 2 trials earlier are different, and a lure condition, under which the stimulus presented one trial earlier is repeated. A crosshair was displayed for 1,000 ms and then the stimulus was presented for 500 ms followed by a 1,000 ms window for response time.

A crosshair was displayed for 1,000 ms and then the stimulus was presented for 500 ms followed by a 1,000 ms window for response time; there was an inter-stimulus interval of 2,500 ms. E-PRIME software (Psychological Software Tools Inc., Sharpsburg, PA, USA) was used for all operations and a block of 30 trials was administered prior to the experimental session to ensure that the participants understood the instructions. The three types of stimuli and the procedure for the stimulus presentation are illustrated in Fig 1.

### Electrophysiological recording procedure

Electrophysiological activity was assessed by electroencephalography (EEG) using a 64-channel Geodesic Sensor net connected to a 64-channel high-input impedance amplifier (Net Amp 300; Electrical Geodesics, Eugene, OR, USA) in a soundproof and electrically shielded experimental room. All electrodes were referenced to Cz and impedance was maintained at 50 kΩ or less [71]. Eye movements and blinks were monitored by electrodes positioned near the outer canthus and beneath the left eye.

All EEG activity was recorded continuously using a 0.01~400 Hz bandpass filter and a sampling rate of 500 Hz; collected EEG data were digitally filtered using a 0.3~30 Hz bandpass filter. Next, the EEG data from the congruent, incongruent, and lure conditions were segmented into 1,000 ms epochs (including a 100 ms pre-stimulus period) that were baseline-corrected. Epochs contaminated by artifacts were removed prior to averaging (based on a threshold of a peak-to-peak amplitude of ± 70 ㎶). Then all remaining data were averaged according to the congruent, incongruent, and lure conditions with an average-reference transformation.

The mean numbers of trials included in the congruent, incongruent, and lure conditions for the BD group were 76.68 (*SD* = 20.20), 156.32 (*SD* = 26.22), and 38.84 (*SD* = 7.90), respectively. The mean numbers of trials included in the congruent, incongruent, and lure conditions for the non-BD group were 77.12 (*SD* = 20.04), 157.00 (*SD* = 25.38), and 37.40 (*SD* = 10.71), respectively. The two groups did not differ significantly in terms of numbers of trials in the congruent (*t*(48) = .04, *p* = .94), incongruent (*t*(48) = .08, *p* = .97), or lure (*t*(48) = -.54, *p* = .59) conditions.

### Statistical analysis

Demographic variables were analyzed with independent *t*-tests. The accuracy rate of the spatial *2*-back task was analyzed with a repeated-measures mixed design analysis of variance (ANOVA) using condition as a within-subject factor (congruent, incongruent, and lure conditions) and group as a between-subject factor (BD and non-BD groups). Response time in the spatial *2*-back task was assessed using independent *t*-tests.

The ERP components and their time windows were determined using the grand-averaged ERPs and individual ERP waveforms. N2 was defined as the most negative peak observed at from 170~340 ms after stimulus-onset; the amplitudes and latencies of N2 were analyzed with a mixed design ANOVA using condition and electrode site (F3, Fz, F4, FC3, FCz, FC4, C3, Cz, and C4) as within-subject factors and group as a between-subject factor. P3 was defined as the most positive peak from 250~550 ms after stimulus-onset; the amplitudes and latencies of P3 were analyzed with a mixed design ANOVA using condition and electrode site (F3, Fz, F4, FC3, FCz, FC4, C3, Cz, C4, P3, Pz, and P4) as within-subject factors and group as a between-subject factor. Green-Geisser corrections were used for violations of sphericity and the corrected *p*-values are reported when appropriate.

## Results

### Demographic characteristics

The BD and non-BD groups did not differ in terms of age (*t*(48) = .24, *p* = .34), educational level (*t*(48) = -.27, *p* = .29), IQ (*t*(48) = 1.22, *p* = .42), SDS (*t*(48) = -1.04, *p* = .69), state anxiety on the STAI (*t*(48) = -.94, *p* = .51), or trait anxiety on the STAI (*t*(48) = -1.78, *p* = .68). However, the BD group exhibited significantly higher AUDIT-K total score (*t*(48) = 18.75, *p* < .001), drinking speed (AUQ item 10) (*t*(48) = 11.13, *p* < .001), number of times being drunk in the previous 6 months (AUQ item 11) (*t*(48) = 6.26, *p* < .001) and percentage of times being drunk when drinking (AUQ item 12) (*t*(48) = 6.37, *p* < .001) compared to the non-BD group. The demographic characteristics of the BD and non-BD groups are presented in Table 1.

**Table 1:** Demographic characteristics of non-binge drinking and binge drinking groups.

	Non-binge drinking (*n* = 25)	Binge drinking (*n* = 25)	*t*
	Mean (SD)	Mean (SD)	
**Age (years)**	22.28 (2.44)	22.12 (2.24)	.24
**Education (years)**	14.92 (1.15)	15.00 (.96)	-.27
**IQ**	116.60 (8.80)	113.84 (7.09)	1.22
**SDS**	41.00 (6.79)	42.92 (6.28	-1.04
**STAI state**	38.88 (11.46)	46.03 (9.38)	-.94
**STAI trait**	40.96 (7.64)	45.08 (8.73)	-1.78
**AUDIT-K**	1.64 (2.02)	18.84 (4.12)	-18.75 ***
**Speed of drinking (drinks/hour)**	.80 (.58)	4.20 (1.41)	-11.13 ***
**Times drunk in the last 6 months**	.20 (.58)	8.36 (6.49)	-6.26 ***
**Percentage of times became drunk when drinking (%)**	10.00 (21.94)	52.80 (25.42)	-6.37***
**Age of starting drinking**	18.15 (.75)	16.96 (1.61)	-3.33**

SDS, Self-Rating Depression Scale; STAI, Spielberger’s State-Trait Anxiety Inventory; AUDIT-K, The Korean version of Alcohol Use Disorder Identify Test; AUQ, Alcohol Use Questionnaire.

***p < .001,

**p < .01

SD, standard deviation

### Behavioral performance on the spatial 2-back task

In terms of accuracy rate, there was a significant main effect of condition (*F*(2,48) = 22.39, *p* < .001) such that the accuracy rates of the lure (*p* < .001) and congruent (*p* < .001) conditions were lower than that of the incongruent condition. There was no significant effect of group (*F*(1,48) = 3.56, *p* = .07) or a significant interaction effect of group × condition (*F*(2,96) = 1.90, *p* = .16). In terms of response time, the main effect of group (*t*(48) = 1.66, *p* = .10) was not significant. The mean accuracy rates and response times of the two groups on the spatial *2*-back task are presented in Table 2.

**Table 2:** Mean accuracy and response time in non-binge drinking and binge drinking groups.

	Non-binge drinking (*n* = 25)			Binge drinking (*n* = 25)		
	Lure	Incongruent	Congruent	Lure	Incongruent	Congruent
**Accuracy (%)**	93	99	94	92	99	90
	(.05)	(.01)	(.06)	(.07)	(.01)	(.11)
**Response time (ms)**			503.28			458.91
			(92.65)			(95.92)

() standard deviation

### Electrophysiological measures

**Fig 2:**
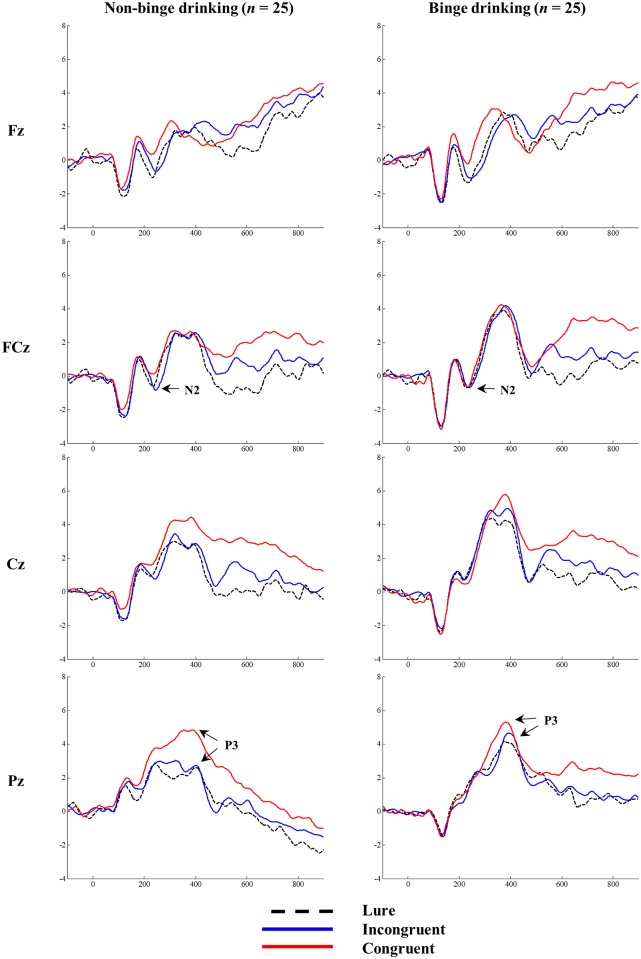
The grand-averaged ERPs. The grand-averaged ERPs elicited by congruent, incongruent, and lure stimuli at Fz, FCz, Cz, and Pz for binge drinking and non-binge drinking groups.

**Fig 3:**
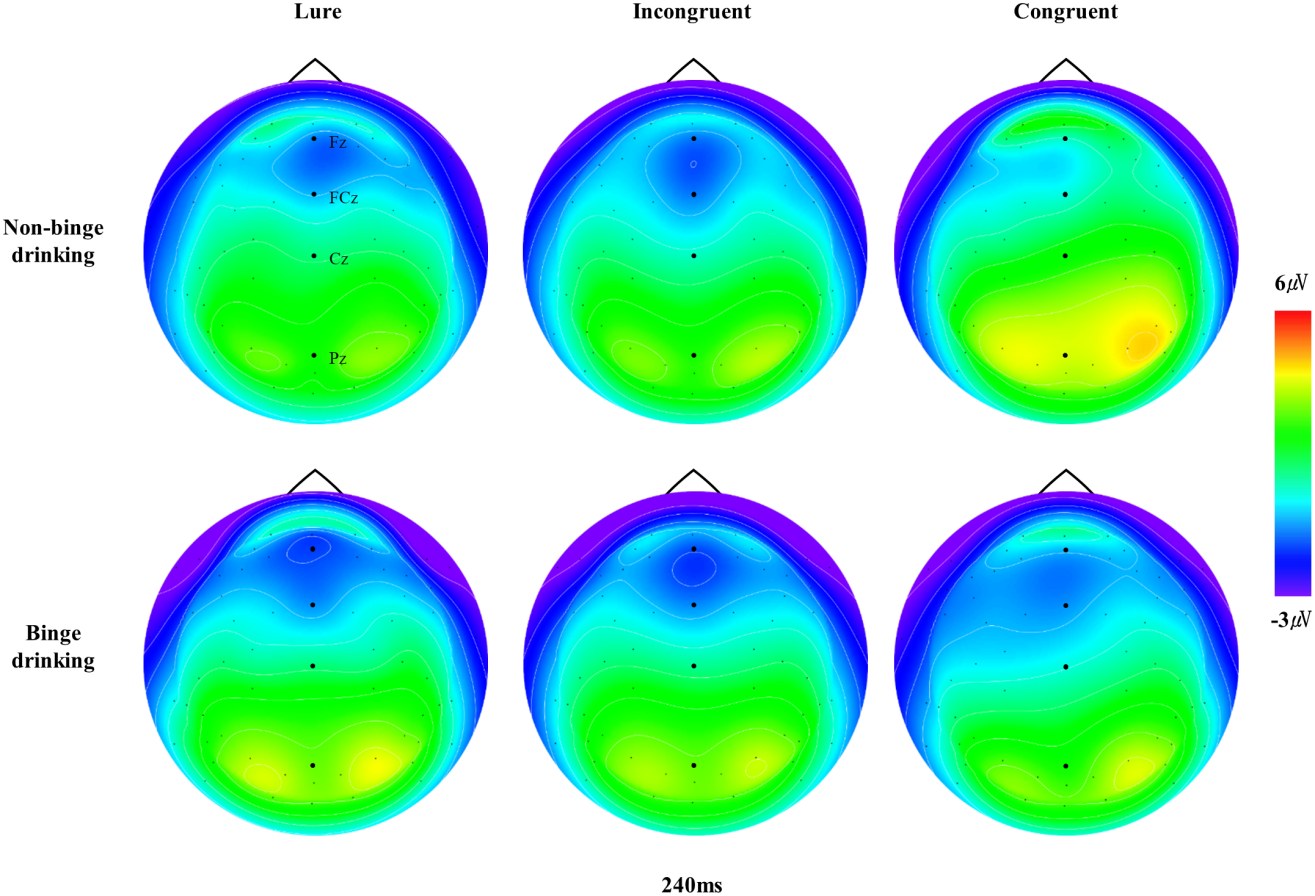
The topographical distributions of N2. The topographical distribution of N2 measured at all electrode sites when the maximum N2 amplitudes were observed.

**Fig 4:**
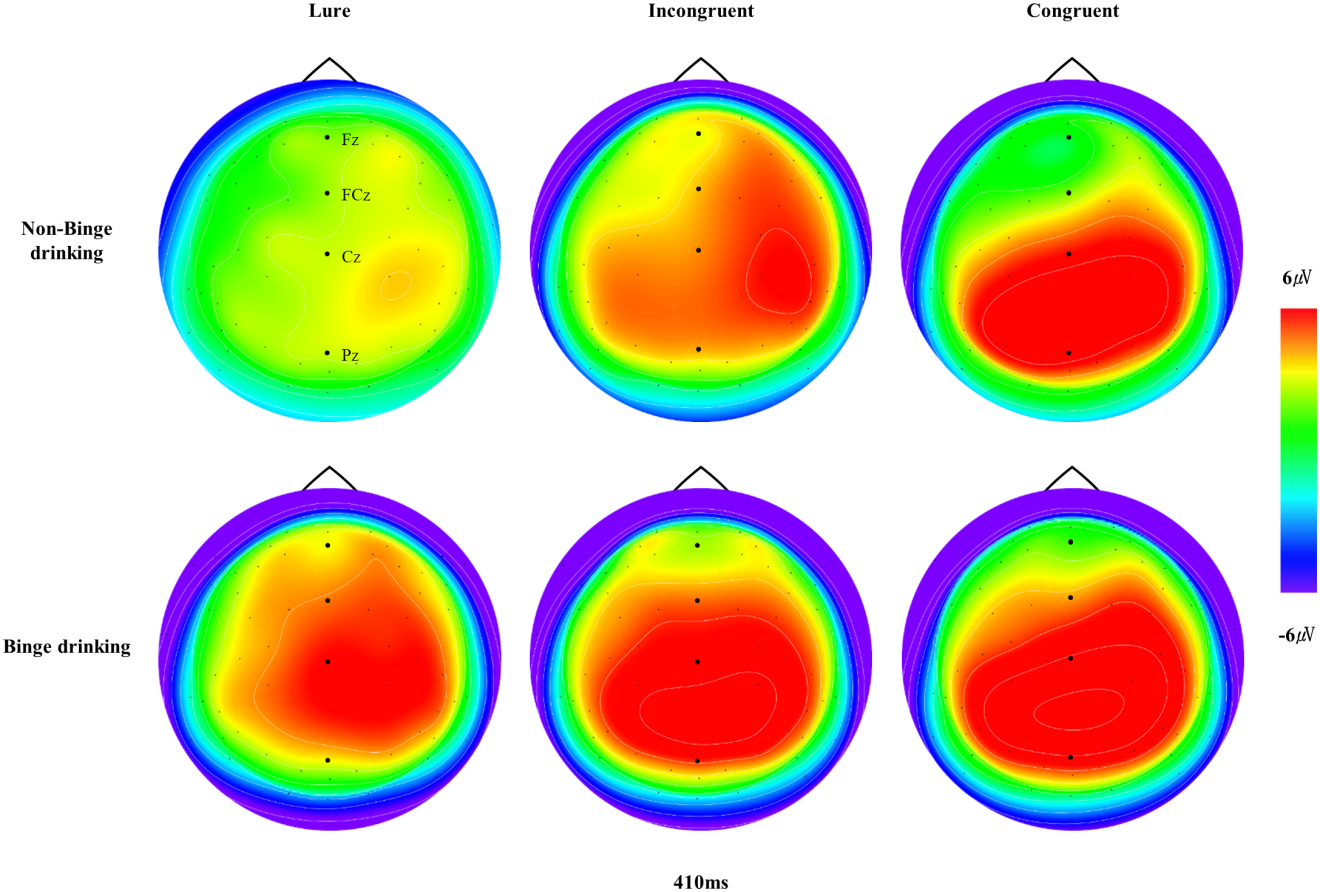
The topographical distributions of P3. The topographical distribution of P3 measured at all electrode sites when the maximum P3 amplitudes were observed.

The grand-averaged ERPs elicited by the lure, incongruent, and congruent stimuli at the midline sites (Fz, FCz, Cz and Pz) for the BD and non-BD groups are displayed in Fig 2. The topographical distributions of N2 and P3 measured at all electrode sites when the maximum N2 and P3 amplitudes were observed are presented in Figs 3 and 4, respectively.

Analyses of the N2 amplitudes revealed significant main effects of condition (*F*(2,96) = 7.57, *p* < .001) and electrode site (*F*(8,384) = 18.49, *p* < .001). The N2 amplitudes in response to the lure (*p* < .01) and incongruent (*p* < .01) stimuli were larger than those for congruent stimuli with the largest amplitude observed at Fz and the smallest observed at C4. There was also a significant interaction effect of condition × electrode site (*F*(16,768) = 4.48, *p* < .001). The N2 amplitudes elicited by the lure and incongruent stimuli were larger than those for congruent stimuli at Fz, F4, and FCz, whereas the N2 amplitudes elicited by the three types of stimuli did not differ at F3, FC3, FC4, C3, or C4. No significant main effect of group (*F*(1,48) = 1.11, *p* = .30) or interaction effects of group × condition (*F*(2,96) = 1.53, *p* = .22) and group × electrode site (*F*(8,384) = .28, *p* = .97) were observed.

In terms of N2 latency, there were significant main effects of condition (*F*(2,96) = 19.41, *p* < .001) and electrode site (*F*(8,384) = 6.24, *p* < .001). The N2 latencies elicited by the lure (*p* < .001) and congruent (*p* < .001) stimuli were significantly shorter than those elicited by incongruent stimuli and the shortest and longest N2 latencies were observed at C4 and F3, respectively. However, no significant main effect of group (*F*(1,48) = .30, *p* = .59) or interaction effects of group × condition (*F*(2,96) = .08, *p* = .92**)** and group × electrode site (*F*(8,384) = .78, *p* = .62) were observed. The mean N2 amplitudes and latencies for the BD and non-BD groups are presented in Table 3.

**Table 3:** Mean N2 amplitudes and latencies in non-binge drinking and binge drinking groups.

	site	Non-binge drinking (*n* = 25)			Binge drinking (*n* = 25)		
		Lure	Incongruent	Congruent	Lure	Incongruent	Congruent
**Amplitude** **(μV)**	**F3**	-0.99	-1.34	-0.57	-1.83	-1.79	-1.39
		(1.97)	(2.09)	(2.17)	(2.13)	(1.83)	(1.90)
	**Fz**	-2.17	-1.40	-0.63	-2.64	-2.18	-1.02
		(2.61)	(2.81)	(2.53)	(2.90)	(2.48)	(2.17)
	**F4**	-1.88	-1.05	-0.47	-2.02	-1.94	-1.22
		(3.06)	(1.92)	(2.03)	(2.18)	(2.39)	(2.15)
	**FC3**	-0.40	-0.39	-0.45	-0.74	-0.82	-1.05
		(1.56)	(1.30)	(1.67)	(1.54)	(1.60)	(1.58)
	**FCz**	-1.92	-1.66	-0.77	-2.04	-1.91	-1.46
		(2.38)	(2.25)	(2.25)	(2.24)	(2.48)	(2.40)
	**FC4**	-0.79	-0.31	0.04	-0.32	-0.79	-0.45
		(1.79)	(1.40)	(1.91)	(1.79)	(1.92)	(1.93)
	**C3**	-0.31	0.01	0.14	-0.44	-0.25	-0.64
		(1.82)	(1.22)	(1.59)	(1.49)	(1.12)	(1.47)
	**Cz**	-0.66	-0.39	0.49	-0.53	-0.45	-0.50
		(2.59)	(1.97)	(2.10)	(1.94)	(1.98)	(2.32)
	**C4**	0.04	0.32	0.63	0.22	-0.31	0.15
		(1.95)	(1.49)	(2.07)	(1.45)	(1.13)	(1.37)
**Latency** **(ms)**	**F3**	242.96	251.12	237.60	240.32	246.08	230.32
		(33.35)	(36.51)	(33.85)	(25.49)	(34.87)	(30.64)
	**Fz**	225.92	242.96	226.32	230.72	248.00	228.48
		(35.09)	(29.20)	(29.00)	(26.04)	(32.10)	(25.62)
	**F4**	235.28	244.08	227.20	238.96	246.80	235.76
		(38.53)	(39.19)	(29.10)	(30.98)	(36.09)	(24.99)
	**FC3**	231.44	240.08	232.56	233.44	246.00	239.60
		(30.87)	(33.72)	(45.83)	(27.76)	(31.53)	(23.44)
	**FCz**	229.60	242.96	234.08	237.60	250.24	233.44
		(29.78)	(34.52)	(36.72)	(27.61)	(31.64)	(36.47)
	**FC4**	225.20	230.24	227.44	235.44	239.36	232.96
		(30.65)	(31.51)	(27.90)	(30.33)	(32.01)	(28.86)
	**C3**	225.84	230.40	222.16	225.28	229.60	226.08
		(38.50)	(38.76)	(30.04)	(30.15)	(35.11)	(25.07)
	**Cz**	233.12	239.04	226.72	229.44	234.80	224.96
		(38.86)	(35.35)	(35.65)	(31.37)	(35.02)	(23.31)
	**C4**	212.16	215.76	211.76	220.16	232.72	217.60
		(29.30)	(31.62)	(35.76)	(30.88)	(39.26)	(30.44)

() standard deviation

In terms of P3 amplitude, there was a significant main effect of group (*F*(1,48) = 7.04, *p* < .05) such that the BD group showed a larger P3 amplitude than the non-BD group. There was also a significant interaction effect of group × condition (*F*(2,96) = 4.24, *p* < .05). The non-BD group showed larger P3 amplitudes in response to the congruent stimuli than in response to the lure (*p* < .001) and incongruent (*p* < .001) stimuli whereas there was no significant difference in P3 amplitude in response to the three types of stimuli in the BD group (*F*(2,48) = 1.24, *p* = .30). There were also significant main effects of condition (*F*(2,96) = 12.84, *p* < .001) and electrode site (*F*(11,528) = 8.93, *p* < .001) such that larger P3 amplitudes were observed in response to the congruent stimuli than in response to the lure (*p* < .001) or incongruent (*p* < .05) stimuli. In terms of electrode site, the largest and smallest P3 amplitudes were observed at Pz and F3, respectively. There was a significant interaction effect of condition × electrode site (*F*(22,1056) = 5.62, *p* < .001) such that larger P3 amplitudes were observed in response to congruent stimuli than in response to lure and incongruent stimuli at Cz, C4, C3, Pz, P3 and P4. The P3 amplitudes elicited by the three types of stimuli did not differ at F3, Fz, F4, FC3, FCz, or FC4.

In terms of P3 latency, there was a significant main effect of condition (*F*(2,96) = 10.47, *p* < .001) with the longest P3 latency observed in response to the incongruent stimuli relative to the lure (*p* < .001) and congruent (*p* < .01) stimuli. However, no significant main effect of group (*F*(1,48) = .48, *p* = .49) or interaction effects of group × condition (*F*(2,96) = .66, *p* = .52**)** and group × electrode site (*F*(11,528) = 1.75, *p* = .08) were observed. The mean P3 amplitudes and latencies for the BD and non-BD groups are presented in Table 4.

**Table 4:** Mean P3 amplitudes and latencies in non-binge drinking and binge drinking groups.

	site	Non-binge drinking (*n* = 25)			Binge drinking (*n* = 25)		
		Lure	Incongruent	Congruent	Lure	Incongruent	Congruent
**Amplitude** **(μV)**	**F3**	2.29	2.53	2.48	4.55	3.97	3.58
		(2.29)	(1.59)	(2.20)	(2.71)	(3.10)	(2.30)
	**Fz**	2.74	3.19	3.55	3.97	3.74	3.67
		(2.53)	(2.37)	(2.71)	(2.84)	(2.81)	(2.29)
	**F4**	3.37	3.24	3.68	4.50	3.92	4.04
		(2.56)	(2.07)	(2.56)	(2.72)	(2.67)	(2.52)
	**FC3**	2.05	2.65	3.01	4.25	4.60	4.38
		(2.29)	(2.21)	(2.19)	(2.64)	(3.06)	(2.47)
	**FCz**	2.53	2.84	3.75	5.04	5.21	4.71
		(2.49)	(2.26)	(2.50)	(3.24)	(3.63)	(3.07)
	**FC4**	3.07	3.60	4.50	4.98	4.89	5.17
		(2.50)	(2.59)	(2.76)	(2.40)	(2.61)	(2.59)
	**C3**	2.54	3.12	3.69	3.99	4.62	4.70
		(2.41)	(2.36)	(2.24)	(1.95)	(2.60)	(2.74)
	**Cz**	2.95	3.37	4.97	5.47	6.07	6.27
		(2.19)	(2.12)	(2.38)	(2.56)	(2.99)	(3.14)
	**C4**	3.57	3.79	4.97	5.12	5.23	5.68
		(2.66)	(2.39)	(2.82)	(2.37)	(2.64)	(2.65)
	**P3**	2.90	3.45	5.06	4.11	5.02	5.59
		(1.70)	(2.46)	(2.68)	(1.64)	(2.06)	(2.28)
	**Pz**	3.79	3.70	5.72	4.52	5.91	6.26
		(2.21)	(1.84)	(1.80)	(1.99)	(2.32)	(2.16)
	**P4**	4.22	4.22	5.53	4.48	5.26	5.77
		(2.42)	(2.27)	(2.81)	(1.79)	(1.93)	(1.96)
**Latency** **(ms)**	**F3**	372.16	371.76	372.48	358.00	376.40	362.88
		(50.67)	(53.65)	(51.21)	(35.39)	(36.66)	(43.26)
	**Fz**	355.52	366.16	365.04	390.40	404.00	377.20
		(46.66)	(55.60)	(68.29)	(34.71)	(44.40)	(50.93)
	**F4**	382.88	399.28	379.04	372.96	392.80	370.56
		(42.67)	(41.56)	(43.60)	(34.51)	(39.56)	(50.39)
	**FC3**	385.84	402.08	390.80	362.96	376.08	376.32
		(46.06)	(37.42)	(40.79)	(36.47)	(45.41)	(43.37)
	**FCz**	376.88	398.24	383.20	378.16	389.76	379.44
		(69.48)	(72.59)	(69.37)	(36.56)	(36.63)	(37.09)
	**FC4**	390.64	394.56	387.44	376.88	385.12	363.12
		(55.29)	(54.11)	(56.92)	(39.75)	(39.96)	(36.58)
	**C3**	375.44	384.40	380.16	378.00	385.68	378.88
		(43.74)	(44.24)	(50.72)	(36.03)	(33.53)	(25.64)
	**Cz**	384.08	388.32	381.92	362.56	371.04	363.44
		(59.30)	(63.65)	(57.71)	(50.79)	(51.13)	(42.53)
	**C4**	372.08	386.72	371.20	385.28	390.96	380.72
		(37.46)	(35.84)	(35.50)	(30.66)	(36.92)	(24.65)
	**P3**	379.76	390.08	386.48	383.60	380.88	378.96
		(44.80)	(38.71)	(43.81)	(44.08)	(43.10)	(29.29)
	**Pz**	377.36	370.00	371.36	371.20	373.84	361.44
		(39.15)	(38.28)	(39.89)	(51.28)	(52.23)	(43.60)
	**P4**	385.92	387.36	380.16	388.72	380.80)	370.96
		(46.33)	(35.42)	(43.33)	(32.32)	(49.49)	(28.74)

() standard deviation

## Discussion

This study investigated spatial working memory in BD college students using ERPs and a modified spatial *2*-back task. The BD and non-BD groups did not differ significantly in terms of response time or accuracy, which is consistent with some [34,35] but not all previous studies [32,72]. These inconsistencies seem to be the result of differences in the participants assessed in these studies. For example, previous studies that reported poor performance in BD individuals included older subjects [72] and subjects who had an earlier onset of alcohol consumption [32] compared to those who participated in this study. In other words, the emergence of behavioral deficits in the working memory task likely required a somewhat long period of BD.

In this study, both groups showed significantly lower accuracy rates and shorter response times in response to the lure and congruent stimuli than in response to the incongruent stimuli. These results indicate that the participants confused the lure stimuli as congruent stimuli which, in turn, resulted in more errors under the congruent and lure conditions than under the incongruent condition [29,30].

In terms of N2 amplitude, both the BD and non-BD groups exhibited larger N2 amplitudes in response to the incongruent and lure stimuli than in response to the congruent stimuli. These results are consistent with those of previous studies showing larger N2 amplitudes in response to incongruent stimuli than in response to congruent stimuli on the *n*-back task [27,29,73]. Additionally, both groups showed significantly shorter response times to congruent and lure stimuli than to incongruent ones, which indicate that the lure stimulus was misidentified as a congruent stimulus. N2 reflects the inhibition of inappropriate responses [29,43] as well as the process involved in detecting whether a current stimulus is the same as or different from one presented *n* trials earlier [42]. The present results (i.e., larger N2 amplitudes in response to incongruent and lure stimuli than in response to congruent stimuli and the longest N2 latency to incongruent stimuli) support these functional activities of N2. In other words, BD individuals maintain the ability to inhibit or detect inappropriate information.

On the other hand, the BD group had significantly larger P3 amplitudes than the non-BD group, and the amplitudes were larger in response to congruent stimuli than in response to incongruent or lure stimuli. Moreover, P3 latency was longer in response to incongruent stimuli than in response to congruent or lure stimuli. The functional significance of P3 amplitude in working memory tasks is not yet fully understood. Some authors have suggested that P3 amplitude reflects the process of classifying task-relevant stimuli [45] whereas others have suggested that it reflects the updating of current information in working memory [46,49,74]. In particular, Kim et al. [75] proposed that a larger P3 amplitude and shorter P3 latency in response to congruent stimuli than in response to incongruent stimuli on the *n*-back task is indicative of the updating of information or of the decision-making process. Still others have suggested that P3 reflects the capacity for attentional allocation [76]. Therefore, enhanced P3 amplitudes in BD individuals may represent enhanced cognitive effort towards the classification and updating of information or attentional allocation to information.

Neuroimaging studies support this interpretation. For example, Campanella et al. [36] measured brain activation in BD and non-BD subjects during the performance of a verbal working memory task and found increased bilateral activation in the pre-supplementary motor area in the BD group relative to the non-BD group despite comparable performances on the verbal working memory task by the two groups. Furthermore, Schweinsburg et al. [77] reported that BD and non-BD groups did not differ in terms of response time or accuracy on spatial working memory tasks but BD individuals exhibited significant increases in activation in the dorsal prefrontal and parietal cortices. These authors suggested that BD individuals maintain performance levels in spatial working memory tasks by increasing activation of brain areas involved in working memory.

In the present study, non-BD individuals showed larger P3 amplitudes in response to congruent stimuli than in response to incongruent and lure stimuli whereas the differences in P3 amplitudes between the congruent and incongruent or lure stimuli were not significant in individuals with BD. These results are consistent with those of previous studies. For example, Crego et al. [55] investigated visual working memory in BD college students and found that non-BD individuals exhibited larger P3 amplitudes in response to congruent stimuli than in response to incongruent stimuli whereas those with BD did not show significant differences in P3 amplitudes between congruent and incongruent stimuli. Taken together, the previous and present results indicate that BD individuals are less efficient in differentiating relevant and irrelevant stimuli and in allocating attentional resources between relevant and irrelevant information.

The present study has several limitations that should be addressed in future studies. First, only a small number of young adult participants was assessed, which limits the generalizability of the present findings. Second, BD individuals are more likely to use other substances, including cigarettes [78], so these substances should be controlled for in future studies. Third, there are significant sex differences in BD in terms of both behavior [32] and electrophysiology [79] and, therefore, future studies should examine whether male and female binge drinkers exhibit different behavioral performances and ERP patterns on working memory tasks. Finally, because the EEG has low spatial resolution in spite of its high temporal resolution, the generators of the N2/P3 could not be clearly identified. Therefore, to elucidate the neurophysiological mechanisms of spatial working memory deficits in BD individuals, neuroimaging techniques or source localization techniques with ERPs should be used in future studies.

In conclusion, the BD and non-BD groups assessed in this study did not differ in terms of response time or accuracy rate on the spatial *2*-back task. However, the BD group exhibited significantly larger P3 amplitudes than the non-BD group and did not show any significant differences in P300 amplitudes among congruent, incongruent, and lure stimuli whereas the non-BD group showed larger P300 amplitudes in response to congruent stimuli than in response to incongruent or lure stimuli. Therefore, the present findings indicate that BD individuals exert greater effort when classifying and updating information or allocating attention to maintain comparable performance on a spatial working memory task relative to non-BD individuals. Furthermore, BD individuals were less efficient in differentiating and allocating attentional resources between relevant and irrelevant information. The results imply that investigations of working memory in BD individuals should employ electrophysiological or neuroimaging techniques as well as behavioral measurements because alteration in the neural network involved in working memory occur prior to the emergence of behavioral deficits. The present results also provide valuable information about the detrimental effects of BD on the neural systems involved in spatial working memory, even when the duration of BD is relatively short (mean months of alcohol consumption in the BD group was 30.92 months).

## Supporting information

S1 File Behavrioral data. (XLSX)

S2 File ERP data. (XLSX)
